# Integrating Biomarkers From Virtual Reality and Magnetic Resonance Imaging for the Early Detection of Mild Cognitive Impairment Using a Multimodal Learning Approach: Validation Study

**DOI:** 10.2196/54538

**Published:** 2024-04-17

**Authors:** Bogyeom Park, Yuwon Kim, Jinseok Park, Hojin Choi, Seong-Eun Kim, Hokyoung Ryu, Kyoungwon Seo

**Affiliations:** 1 Department of Applied Artificial Intelligence Seoul National University of Science and Technology Seoul Republic of Korea; 2 Department of Neurology College of Medicine Hanyang University Seoul Republic of Korea; 3 Graduate School of Technology and Innovation Management Hanyang University Seoul Republic of Korea

**Keywords:** magnetic resonance imaging, MRI, virtual reality, VR, early detection, mild cognitive impairment, multimodal learning, hand movement, eye movement

## Abstract

**Background:**

Early detection of mild cognitive impairment (MCI), a transitional stage between normal aging and Alzheimer disease, is crucial for preventing the progression of dementia. Virtual reality (VR) biomarkers have proven to be effective in capturing behaviors associated with subtle deficits in instrumental activities of daily living, such as challenges in using a food-ordering kiosk, for early detection of MCI. On the other hand, magnetic resonance imaging (MRI) biomarkers have demonstrated their efficacy in quantifying observable structural brain changes that can aid in early MCI detection. Nevertheless, the relationship between VR-derived and MRI biomarkers remains an open question. In this context, we explored the integration of VR-derived and MRI biomarkers to enhance early MCI detection through a multimodal learning approach.

**Objective:**

We aimed to evaluate and compare the efficacy of VR-derived and MRI biomarkers in the classification of MCI while also examining the strengths and weaknesses of each approach. Furthermore, we focused on improving early MCI detection by leveraging multimodal learning to integrate VR-derived and MRI biomarkers.

**Methods:**

The study encompassed a total of 54 participants, comprising 22 (41%) healthy controls and 32 (59%) patients with MCI. Participants completed a virtual kiosk test to collect 4 VR-derived biomarkers (hand movement speed, scanpath length, time to completion, and the number of errors), and T_1_-weighted MRI scans were performed to collect 22 MRI biomarkers from both hemispheres. Analyses of covariance were used to compare these biomarkers between healthy controls and patients with MCI, with age considered as a covariate. Subsequently, the biomarkers that exhibited significant differences between the 2 groups were used to train and validate a multimodal learning model aimed at early screening for patients with MCI among healthy controls.

**Results:**

The support vector machine (SVM) using only VR-derived biomarkers achieved a sensitivity of 87.5% and specificity of 90%, whereas the MRI biomarkers showed a sensitivity of 90.9% and specificity of 71.4%. Moreover, a correlation analysis revealed a significant association between MRI-observed brain atrophy and impaired performance in instrumental activities of daily living in the VR environment. Notably, the integration of both VR-derived and MRI biomarkers into a multimodal SVM model yielded superior results compared to unimodal SVM models, achieving higher accuracy (94.4%), sensitivity (100%), specificity (90.9%), precision (87.5%), and *F*_1_-score (93.3%).

**Conclusions:**

The results indicate that VR-derived biomarkers, characterized by their high specificity, can be valuable as a robust, early screening tool for MCI in a broader older adult population. On the other hand, MRI biomarkers, known for their high sensitivity, excel at confirming the presence of MCI. Moreover, the multimodal learning approach introduced in our study provides valuable insights into the improvement of early MCI detection by integrating a diverse set of biomarkers.

## Introduction

### Background

Mild cognitive impairment (MCI) represents a transitional stage in cognitive decline, positioned between normal aging and Alzheimer disease (AD). This stage is marked by challenges such as memory loss and difficulties in executing complex daily activities [1-3]. Once MCI deteriorates into AD, cognitive function cannot be restored to normal levels [4,5], leading to a significant reduction in the ability of patients with AD to independently perform daily activities [6]. This makes MCI a critical intervention point to potentially slow down cognitive decline. Therefore, the early detection of MCI plays an essential role, not only in preventing its progression to AD but also in allowing for timely interventions aimed at restoring cognitive function to levels associated with normal aging [7].

Conventionally, biomarkers such as neuropsychological tests and magnetic resonance imaging (MRI) have been deployed for early detection of MCI, facilitating the evaluation of cognitive functions and the tracking of brain changes [8]. For instance, neuropsychological tests aim to quantitatively assess multiple cognitive domains, including memory, to identify patients with MCI [9]. However, despite their widespread clinical use, these tests face challenges related to reproducibility, largely owing to elements such as response bias and the ceiling effect [10]. In contrast, MRI scans identify MCI by examining structural brain changes, particularly in memory-associated regions such as the hippocampus and entorhinal cortex [11,12]. However, the use of MRI scans is restricted due to their limited feasibility for regular and repeated assessments, given their lengthy procedures and high costs [13,14]. Thus, there is an increasing need for novel biomarkers that can effectively and economically detect MCI by leveraging reproducible behavioral characteristics observed in daily activities [15-18].

In recent studies, virtual reality (VR) technology has been used to collect behavioral data related to instrumental activities of daily living (IADLs), which are then analyzed using machine learning techniques to enhance the early detection of MCI [17-20]. As an example, Kim et al [21] devised the virtual kiosk test, wherein participants interact within a virtual environment to order food using a kiosk. Throughout this test, behavioral data related to hand movements, eye movements, and performance were collected. Using these behavioral data, the machine learning model successfully distinguished patients with MCI from healthy controls with an accuracy rate of 80.2%. Although VR-derived biomarkers have shown promise in early MCI detection, there is still an ongoing question regarding the interpretation of these biomarkers in relation to measurable structural brain changes observed through MRI, which is of critical clinical significance.

The integration of behavioral characteristics measured through VR-derived biomarkers with the brain’s structural characteristics obtained from MRI biomarkers is gaining increasing attention. For example, Castegnaro et al [22] examined object location memory using VR performance data and found that individuals with damage to the hippocampus and entorhinal cortex demonstrated poorer performance, suggesting a correlation between impaired performance and damage to these brain regions. Similarly, Howett et al [23] conducted a VR navigation task and observed that participants with entorhinal cortex damage exhibited inferior performance in the task. Although these studies established significant positive correlations between performance assessed using VR-derived biomarkers and atrophy in the hippocampus and entorhinal cortex identified using MRI biomarkers, the results were confined to correlation analysis. To address this limitation, Cavedoni et al [19] emphasized the importance of further exploring the multimodal integration of VR-derived and MRI biomarkers, as it would not only establish the clinical validity of VR but also provide valuable behavioral and structural information about MCI. Consistent with these objectives, this study aimed to leverage a multimodal learning approach by integrating VR-derived and MRI biomarkers to enhance clinical feasibility and enable early and accurate detection of MCI.

### Objectives

This study had 2 objectives. First, the study compared the MCI classification performance of VR-derived and MRI biomarkers with that of neuropsychological tests, which are considered the gold standard for MCI classification. This comparative analysis aimed to provide a deeper understanding of the advantages and limitations of each approach, namely VR, MRI, and neuropsychological tests. Second, the study introduced and validated a multimodal learning model that effectively improves the early detection of MCI by integrating the unique attributes of VR-derived and MRI biomarkers. This integrated approach harnesses the strengths of both modalities for more accurate and reliable MCI detection. The findings of this study proposed a novel clinical application approach that incorporates VR-derived and MRI biomarkers sequentially, offering a promising framework for clinicians to enhance their diagnostic capabilities in evaluating MCI.

## Methods

### Ethical Considerations

This study received ethics approval from the institutional review board of Hanyang University Hospital, Republic of Korea, in accordance with the principles outlined in the Declaration of Helsinki (HYUH-2021-08-020-004). Before conducting the experiment, we ensured that all participants received a comprehensive explanation of the study, and we obtained written informed consent from each individual. In addition, all participants provided consent for the use of their de-identified data for scientific research purposes, and only such data were used in the subsequent analysis. As a token of appreciation for their participation, participants were offered compensation in the form of detailed reports summarizing the results from neuropsychological tests, VR tasks, and MRI.

### Participants

This study involved a total of 54 participants recruited from Hanyang University Hospitals in Seoul and Guri between January 2022 and July 2023. The 54 participants included 27 (50%) individuals from Seoul (n=11, 41% healthy controls and n=16, 59% patients with MCI) and 27 (50%) individuals from Guri (n=11, 41% healthy controls and n=16, 59% patients with MCI). Participants were randomly recruited from both a volunteer pool and outpatient clinics at the hospitals. The 2 neurologists reached a consensus on the diagnosis of MCI based on the cutoff score of the Seoul Neuropsychological Screening Battery–Core (SNSB-C), a gold-standard neuropsychological assessment tool specifically standardized for the Korean population [24]. The study only included participants who were capable of interacting with the VR environment and who possessed normal sensory perception, specifically in response to visual and auditory stimuli. We excluded individuals with a history of dementia, neurodegenerative disorders, or psychiatric conditions or those who had undergone brain surgeries. It is important to note that one volunteer (1/27, 4%) from Guri was excluded from recruitment due to a history of brain surgery for hydrocephalus. None of the patients with MCI included in the study were diagnosed with dementia.

### Neuropsychological Tests

This study used the SNSB-C, a neuropsychological test specifically designed and standardized for the Korean population. The SNSB-C serves as a reliable and clinically validated alternative to the Mini‐Mental State Examination for evaluating cognitive functions [24,25]. It assesses five distinct cognitive domains through separate assessments, including the following: (1) attention—measured using the Digit Span Test–Backward, (2) language function—evaluated using the Short Form of the Korean-Boston Naming Test, (3) visuospatial function—assessed using the Rey Complex Figure Test (RCFT), (4) memory—measured using the Seoul Verbal Learning Test–Elderly’s Version–Delayed Recall, and (5) frontal and executive function—evaluated using the Digit Symbol Coding. A professional psychological evaluator with 15 years of experience conducted all the SNSB-C evaluations in this study.

### VR-Derived Biomarkers

**Figure 1:**
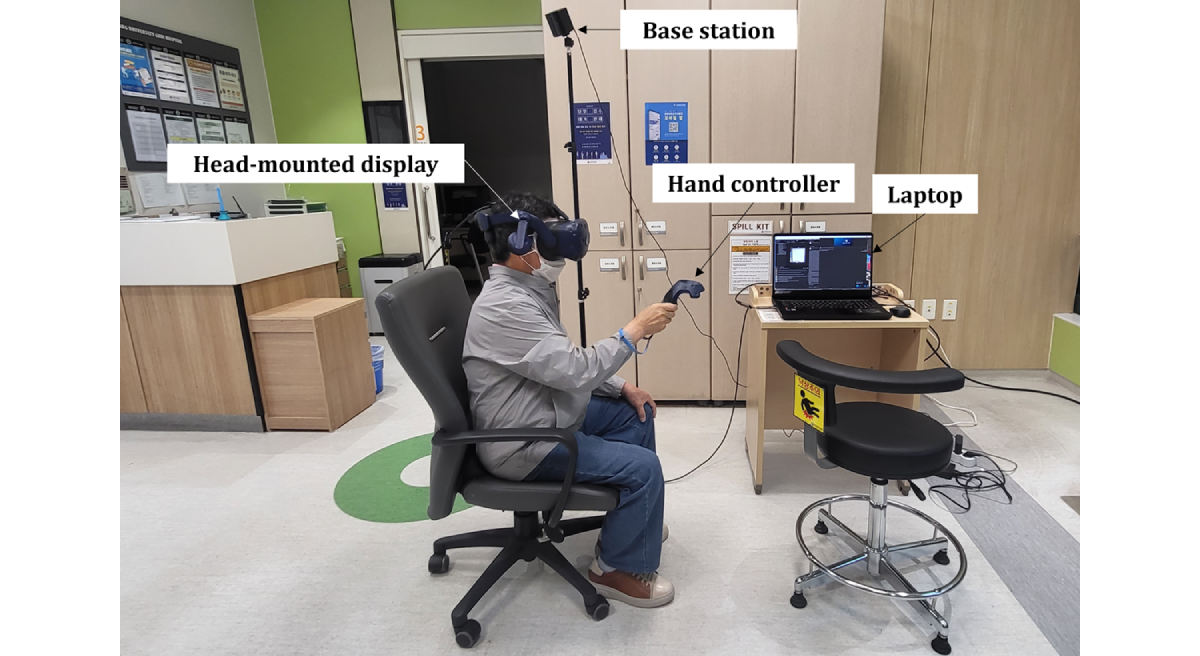
Experimental setup for the virtual kiosk test. The virtual kiosk test is operated via a laptop. Participants sit and wear a head-mounted display and interact with the virtual environment using a hand controller. Their hand movements, eye movements, and performance data are tracked via base stations.

This study used the virtual kiosk test, previously developed in our research [26], as a source of VR-derived biomarkers. VR-derived biomarkers consist of features used to assess cognitive impairment by analyzing behavioral data, including hand and eye movements, collected from VR environments [17]. The virtual kiosk test aims to detect early indications of MCI by assessing behavioral data collected while participants undertake a cognitively complex IADL task, specifically ordering menu items at a virtual kiosk. The experimental setup, as shown in Figure 1, was arranged in a room containing a laptop equipped with an Intel i7-12700H processor, 16 GB of RAM, and an NVIDIA GeForce RTX 3080 graphics card to execute Unity and VIVEPORT software, which is necessary to run the VR program. To facilitate a fully immersive VR experience, participants wore a head-mounted display with integrated eye-tracking capabilities (HTC VIVE Pro Eye) and used a hand controller in their dominant hand to select and order menu items from the virtual kiosk. Overall, 2 base stations tracked participants’ movements during the test. The behavioral data recorded during this task, encompassing hand and eye movements and performance data, can provide insightful VR-derived biomarkers. For safety, participants remained seated throughout the test.

**Figure 2:**
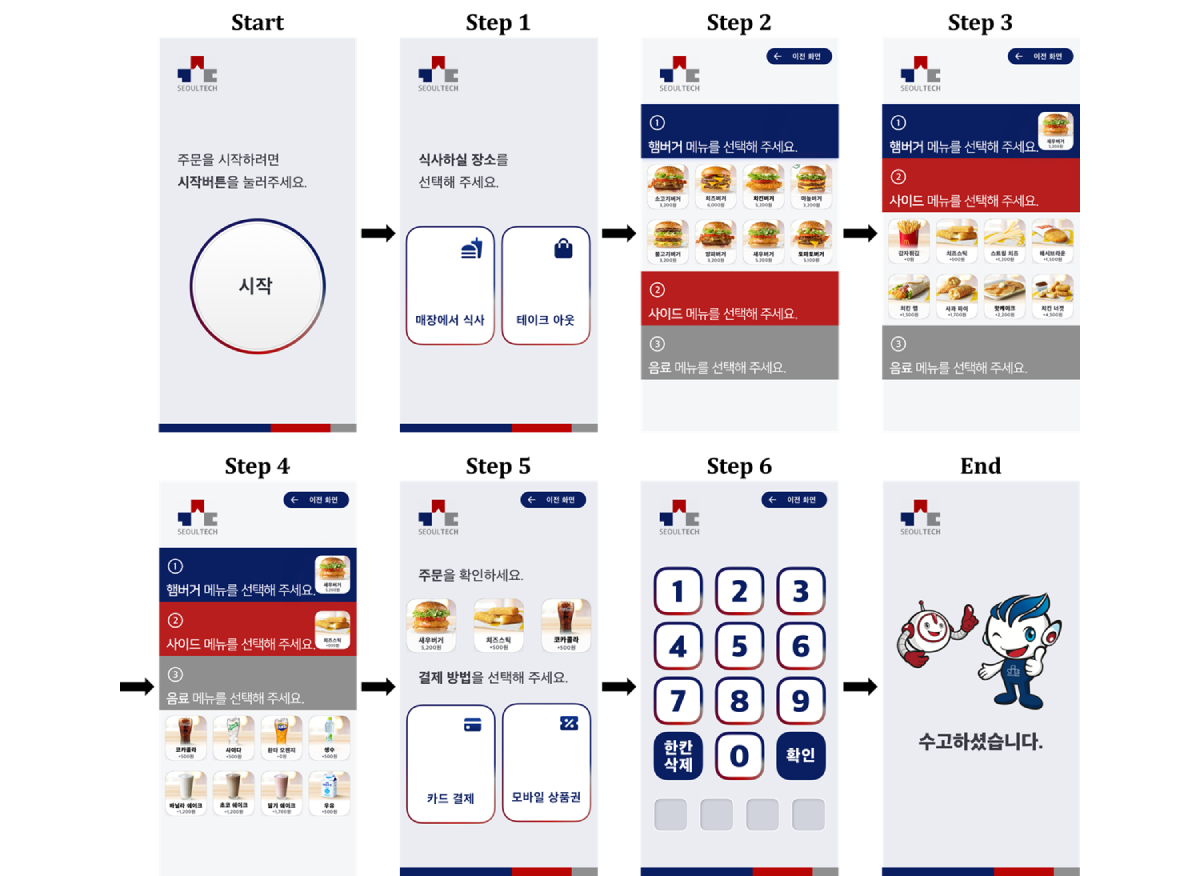
The 6 sequential steps of the virtual kiosk test. In step 1, participants selected a place to eat. In step 2, they chose a burger item. Step 3 involved selecting a side item, and in step 4, participants chose a drink item. Step 5 required them to select a payment method, and finally, in step 6, they entered the payment password.

The virtual kiosk test, depicted in Figure 2, followed six steps, not including “Start” and “End” stages: (1) selecting a place to eat, (2) choosing a burger item, (3) selecting a side item, (4) choosing a drink item, (5) selecting a payment method, and (6) entering a 4-digit payment password. Before the test, participants received the following instructions verbally: “The place to eat is a restaurant. Please use the kiosk to order a shrimp burger, cheese sticks, and a Coca-Cola. Use a credit card as the payment method, and the card payment password is 6289.”

**Figure 3:**
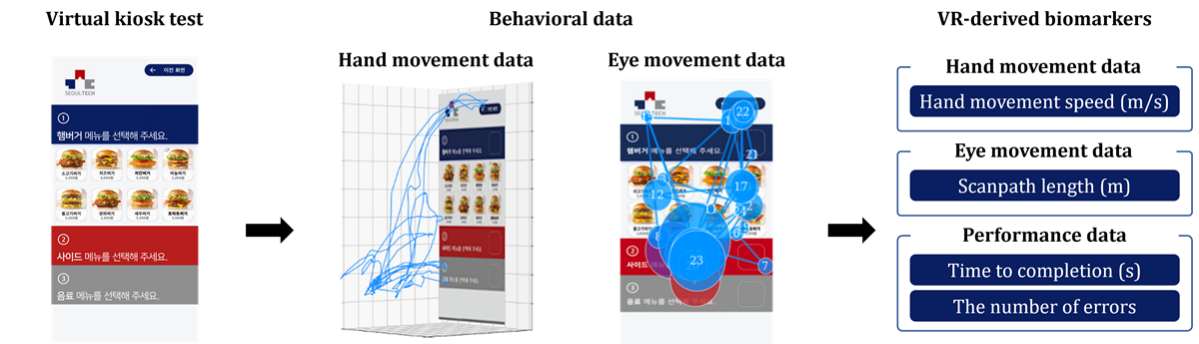
Extraction of 4 virtual reality (VR)–derived biomarkers from behavioral data in the virtual kiosk test. Hand movement speed is calculated using the hand movement data collected from the virtual kiosk test. Scanpath length is derived from the eye movement data. The time to completion and the number of errors are calculated based on the performance data.

Then, the behavioral data collected during the test were converted into 4 VR-derived biomarkers: hand movement speed, scanpath length, time to completion, and the number of errors (Figure 3). Hand movement speed, the first VR-derived biomarker, was determined by dividing the total distance of hand movements by the total test time, which correlates with cognitive abilities such as recognition and processing speed [21,27-29]. Scanpath length, the second VR-derived biomarker, reflects the distance traveled by participants’ gaze during the test, indicating overall cognitive ability [30], information processing efficiency [31], and comprehension level [32]. Time to completion [33], the third VR-derived biomarker, represents the duration required for participants to complete all 6 steps of the test. The number of errors [26], the final VR-derived biomarker, records the total number of incorrect actions during the test, such as incorrect choices for place to eat, burger, drink, side item, or payment method or incorrect password entry.

### MRI Biomarkers

MRI scans were performed at Hanyang University Hospital in both Guri and Seoul locations using a Philips Ingenia CX 3T scanner. The scans used the 3D T_1_-weighted magnetization-prepared rapid gradient echo technique, with specific protocols set for each hospital. The hospital at Guri adhered to the following parameters: echo time/repetition time=2.9 ms/6.3 ms, flip angle=9°, field of view=256×256 mm, with 211 slices and voxel size of 1×1×1 mm^3^. The hospital at Seoul followed a slightly different protocol: echo time/repetition time=4.1 ms/6.9 ms, flip angle=8°, field of view=300×299 mm, with 170 slices and voxel size of 0.8×0.8×1 mm^3^.

**Figure 4:**
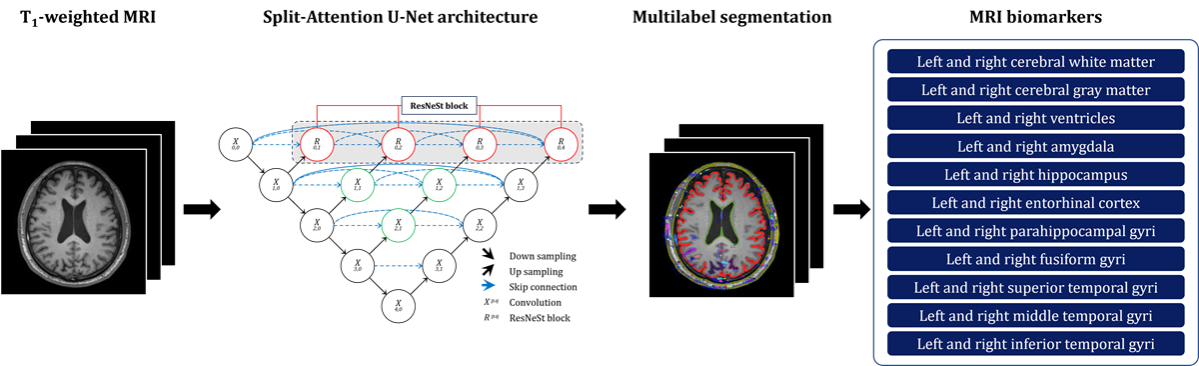
Extraction of 22 magnetic resonance imaging (MRI) biomarkers from both hemispheres of the brain using the Split-Attention U-Net architecture. Following multilabel segmentation of the region of interest in the brain, each brain region’s volume is quantified as an MRI biomarker. Each hemisphere has 11 biomarkers including the cerebral white matter; cerebral gray matter; ventricles; amygdala; hippocampus; entorhinal cortex; parahippocampal gyrus; fusiform gyrus; and superior, middle, and inferior temporal gyrus.

We used AQUA 3.0 software (Neurophet Inc) to process the acquired T_1_-weighted MRIs, a method that aids in precise identification and outlining of the region of interest in the brain as depicted in Figure 4 [34,35]. The AQUA 3.0 software uses the Split-Attention U-Net deep learning architecture, which combines elements from ResNeSt and U-Net++. The architecture of Split-Attention U-Net demonstrates robustness to neuroanatomical variability through its encoder and decoder structure, skip pathways, and split-attention module. It also improves segmentation accuracy, particularly for small subcortical regions, by using EvoNorm-based convolution layers and 3D ResNeSt blocks. This methodological approach provides valuable information about brain volume from MRIs, which can be used as MRI biomarkers for neuroimaging analysis. Furthermore, the software accounts for cerebral hemispheric asymmetry by separately analyzing the left and right hemispheres, thus enabling a more holistic evaluation.

In this study, we selected specific MRI biomarkers associated with brain regions known to exhibit early atrophy in AD and play crucial roles in various cognitive functions [12,36]. The chosen MRI biomarkers, as described in Table 1, encompass cerebral white matter [37]; cerebral gray matter [38]; ventricles [39]; amygdala [40]; hippocampus [41,42]; entorhinal cortex [41,43]; parahippocampal gyrus [43]; fusiform gyrus [44]; and the superior, middle, and inferior temporal gyri [45]. These MRI biomarkers were derived from both hemispheres, resulting in a total of 22 MRI biomarkers used in this study. Furthermore, we incorporated intracranial volume (ICV), a measure encapsulating total white matter, gray matter, and cerebrospinal fluid [46], to account for overall brain size. Then, we normalized all the MRI biomarkers to the ICV to mitigate differences in brain volume due to factors such as age, sex, and head circumference [14].

**Table 1:** The 11 magnetic resonance imaging biomarkers for each hemisphere (ie, a total of 22 magnetic resonance imaging biomarkers from both hemispheres) and their descriptions.

Biomarkers	Description
Cerebral white matter	Cerebral white matter is associated with brain functions such as learning, memory, and transmitting neural information between brain regions.
Cerebral gray matter	Cerebral gray matter is associated with cognitive functions, encompassing information processing, decision-making, and sensory perception.
Ventricles	Ventricles are associated with cerebrospinal fluid circulation, maintaining optimal conditions for cognitive processes in the brain.
Amygdala	The amygdala is associated with emotional processing, and the modulation of memory consolidation.
Hippocampus	The hippocampus is associated with memory formation, spatial memory, and emotional processing.
Entorhinal cortex	The entorhinal cortex is associated with functioning as a key network for spatial memory and the perception of time.
Parahippocampal gyrus	The parahippocampal gyrus is associated with cognitive processes such as spatial and episodic memory.
Fusiform gyrus	The fusiform gyrus is associated with high-level vision and multisensory perception such as object and word recognition.
Superior temporal gyrus	The superior temporal gyrus is associated with the analysis of audio-visual social information such as verbal and nonverbal communication.
Middle temporal gyrus	The middle temporal gyrus is associated with language-related tasks and integration of audio-visual emotional processing.
Inferior temporal gyrus	The inferior temporal gyrus is associated with object recognition, such as recognizing objects based on prior experiences.

### Procedures

Participants initially underwent an assessment using the SNSB-C, administered by a professional psychological evaluator with 15 years of experience. MCI diagnosis was subsequently performed by 2 experienced neurologists with 18 years and 22 years of clinical expertise, adhering to the criteria established by Albert et al [47], which considered the results of the SNSB-C. Then, participants completed both the virtual kiosk test and T_1_-weighted MRI scans in counterbalanced order. The virtual kiosk test was administered by the same neurologists responsible for the MCI diagnosis, whereas T_1_-weighted MRI scans were conducted by a radiologist with 16 years of experience. To ensure participants’ comfort and familiarity with the VR setup, they underwent 2 practice sessions before the virtual kiosk test, allowing them to get accustomed to the VR equipment and virtual environment. Throughout the experiment, measures were in place allowing participants to take breaks or halt the procedure if they experienced discomfort or dizziness due to either the VR environment or MRI scans. It is important to note that all participants completed the entire experiment successfully in an average time of 54.32 (SD 4.27) minutes, without requiring any breaks.

### Analysis

We conducted statistical analysis using SPSS Statistics (version 27; IBM Corp). First, we compared of the study participants, encompassing aspects such as sex, age, and education level, were examined using a chi-square test and independent sample *t* tests to identify any statistically significant differences between the healthy control group and patients with MCI group, using a *χ*^2^ test for a categorical variable (ie, sex) and independent sample 2-tailed *t* tests for continuous variables. Second, we performed analyses of covariance (ANCOVA) to assess the differences in neuropsychological characteristics, VR-derived biomarkers, and MRI biomarkers between the healthy controls and patients with MCI, while considering age as a covariate in this analysis. This allowed us to discern distinct features within each biomarker. In addition, we examined the relationship between VR-derived and MRI biomarkers by conducting a Pearson correlation analysis. Notably, as our data exhibited a normal distribution and homoscedasticity, all analyses were conducted using parametric tests.

### Multimodal Learning

This study used multimodal learning using Python 3, with the ultimate objective of amalgamating statistically significant VR-derived and MRI biomarkers to bolster early MCI detection. The support vector machine (SVM) algorithm, with a track record of extensive use and demonstrated effectiveness in analogous tasks [4,48-50], was chosen as the machine learning model for this study. Hyperparameters for the SVM algorithm were identified through grid search, resulting in the choice of the radial basis function kernel with the regularization parameter (cost) set at 1 and kernel coefficient (γ) set at 0.1. For external validation and to prevent overfitting, we used a train and test split with a ratio of 7:3, where 70% (38/54) of the participants were assigned to the *train* subcohort and the remaining 30% (16/54) of the participants were allocated to the *test* subcohort. During the biomarker integration process, the comparative performance of models using individual VR-derived and MRI biomarkers was examined, revealing the unique characteristics of each modality. The performance evaluation of these models was conducted using several metrics, including accuracy, sensitivity, specificity, precision, and *F*_1_-score. In addition, the area under the receiver operating characteristic curve (AUC) was used to evaluate the performance of the binary classifier (healthy controls vs patients with MCI) in our models.

## Results

### Demographic and Neuropsychological Characteristics

The demographic characteristics of the study participants, encompassing aspects such as sex, age, and education level, were examined using a chi-square test and independent sample *t* tests to identify any statistically significant differences between the healthy control group and patients with MCI group. As per the results outlined in Table 2, no significant differences were found in terms of demographic characteristics between the 2 groups. Subsequently, neuropsychological characteristics were assessed using ANCOVA, with age as a covariate. The results showed a distinct contrast between the healthy control group and patients with MCI group. Patients with MCI exhibited significant impairment across all 5 evaluated cognitive functions—attention (*F*_1,51_=24.181; *P*<.001; η_p_^2^=0.322), language function (*F*_1,51_=14.993; *P*<.001; η_p_^2^=0.227), visuospatial function (*F*_1,51_=19.115; *P*<.001; η_p_^2^=0.273), memory (*F*_1,51_=32.542; *P*<.001; η_p_^2^=0.390), and frontal and executive function (*F*_1,51_=20.584; *P*<.001; η_p_^2^=0.288)—when compared to the healthy control group, thus underscoring a marked cognitive decline in individuals with MCI.

**Table 2:** Comparison of basic demographic characteristics and neuropsychological test results between healthy controls and patients with mild cognitive impairment (MCI).

Characteristics			Group					*P* value
			Healthy controls (n=22)		Patients with MCI (n=32)			
**Basic demographic characteristics**								
	Sex (female), n (%)	14 (64)		14 (44)		.15		
	Age (y), mean (SD)	69.86 (6.72)		73.47 (8.39)		.07		
	Educational level (years), mean (SD)	12.09 (4.46)		9.47 (5.12)		.06		
**Neuropsychological tests, mean (SD)**								
	DST–B^a^ (number of correct answers)	4.27 (0.83)		3.00 (0.88)		<.001^b^		
	S-K–BNT^c^ (number of correct answers)	12.91 (1.44)		10.78 (2)		<.001^b^		
	RCFT^d^ (score)	33.34 (2.31)		26.95 (5.93)		<.001^b^		
	SVLT-E–DR^e^ (number of correct answers)	6.82 (2.63)		2.50 (2.55)		<.001^b^		
	DSC^f^ (number of correct answers)	60.95 (15.83)		37.97 (17.24)		<.001^b^		

^a^DST–B: Digit Span Test–Backward.

^b^Analyses of covariance, with age as a covariate.

^c^S-K–BNT: Short Form of the Korean-Boston Naming Test.

^d^RCFT: Rey Complex Figure Test.

^e^SVLT-E–DR: Seoul Verbal Learning Test–Elderly’s version–Delayed Recall.

^f^DSC: Digital Symbol Coding.

### Differences in VR-Derived Biomarkers Between Healthy Controls and Patients With MCI

As illustrated in Table 3, significant differences were observed in VR-derived biomarkers between the healthy controls and patients with MCI when assessed through ANCOVA, with age as a covariate. Specifically, patients with MCI demonstrated slower hand movement speed (*F*_1,51_=13.426; *P*=.001; η_p_^2^=0), longer scanpath length (*F*_1,51_=7.108; *P*=.01; η_p_^2^=0.122), prolonged time to completion (*F*_1,51_=9.447; *P*=.003; η_p_^2^=0.156), and a greater number of errors (*F*_1,51_=9.438; *P*=.003; η_p_^2^=0.156) during the execution of the virtual kiosk test, as compared to healthy controls.

**Table 3:** Comparison of virtual reality (VR)–derived biomarkers between healthy controls and patients with mild cognitive impairment (MCI).

VR-derived biomarkers			Group, mean (SD)				*P* value^a^
			Healthy controls (n=22)		Patients with MCI (n=32)		
**Hand movement features**							
	Hand movement speed (m/s)	0.23 (0.06)		0.17 (0.06)		.001	
**Eye movement feature**							
	Scanpath length (m)	23.66 (14.29)		60.36 (54.58)		.01	
**Performance features**							
	Time to completion (s)	39.48 (18.96)		105.39 (86.35)		.003	
	Number of errors	1.73 (1.61)		4 (2.81)		.003	

^a^Analyses of covariance, with age as a covariate.

### Differences in MRI Biomarkers Between Healthy Controls and Patients With MCI

ANCOVA was performed with age as a covariate to scrutinize the discrepancies in ICV and the proportion of ICV between the healthy control group and the patients with MCI group. As presented in Table 4, although patients with MCI exhibited higher ICV compared to healthy controls, this difference was not statistically significant. While higher ICV is typically associated with age and sex effects [51-53], our statistical analysis did not identify significant variations in age and sex between healthy controls and patients with MCI. This suggests that factors other than age and sex may be contributing to the observed higher ICV among our patients with MCI. However, patients with MCI demonstrated notable atrophy in the proportion of ICV. Specifically, significant differences were discerned in the left entorhinal cortex (*F*_1,51_=7.821; *P*=.007; η_p_^2^=0.133), right entorhinal cortex (*F*_1,51_=11.103; *P*=.002; η_p_^2^=0.179), left hippocampus (*F*_1,51_=11.926; *P*=.001; η_p_^2^=0.190), right hippocampus (*F*_1,51_=8.244; *P*=.006; η_p_^2^=0.139), left amygdala (*F*_1,51_=7.979; *P*=.007; η_p_^2^=0.135), and right amygdala (*F*_1,51_=6.618; *P*=.01; η_p_^2^=0.115). Refer to Multimedia Appendix 1 for detailed raw volumes of MRI biomarkers comparing healthy controls and patients with MCI.

**Table 4:** Comparison of magnetic resonance imaging (MRI) biomarkers between healthy controls and patients with mild cognitive impairment (MCI).

MRI biomarkers		Group, mean (SD)			*P* value^a^
		Healthy controls (n=22)	Patients with MCI (n=32)		
**Raw volume (cc)**					
	ICV^b^	1490.96 (127.40)	1511.49 (128.89)	.56	
**The proportion of ICV (%)**					
	Left cerebral white matter	14.40 (0.73)	14.06 (0.91)	.62	
	Right cerebral white matter	14.51 (0.70)	14.13 (0.93)	.48	
	Left cerebral gray matter	15.52 (1.01)	14.98 (0.91)	.13	
	Right cerebral gray matter	15.46 (1.10)	14.77 (1.01)	.06	
	Left ventricles	1.28 (0.70)	1.57 (0.84)	.48	
	Right ventricles	1.16 (0.80)	1.41 (0.76)	.52	
	Left amygdala	0.11 (0.01)	0.10 (0.01)	.007	
	Right amygdala	0.12 (0.01)	0.11 (0.01)	.002	
	Left hippocampus	0.24 (0.02)	0.21 (0.03)	.001	
	Right hippocampus	0.24 (0.02)	0.22 (0.03)	.006	
	Left entorhinal cortex	0.15 (0.02)	0.13 (0.02)	.007	
	Right entorhinal cortex	0.13 (0.01)	0.11 (0.02)	.01	
	Left parahippocampal gyrus	0.13 (0.02)	0.12 (0.02)	.23	
	Right parahippocampal gyrus	0.12 (0.02)	0.12 (0.02)	.26	
	Left fusiform gyrus	0.60 (0.06)	0.58 (0.06)	.39	
	Right fusiform gyrus	0.57 (0.07)	0.57 (0.06)	.78	
	Left superior temporal gyrus	0.72 (0.07)	0.69 (0.06)	.17	
	Right superior temporal gyrus	0.69 (0.06)	0.67 (0.06)	.64	
	Left middle temporal gyrus	0.72 (0.08)	0.66 (0.08)	.11	
	Right middle temporal gyrus	0.69 (0.08)	0.65 (0.08)	.09	
	Left inferior temporal gyrus	0.76 (0.06)	0.74 (0.06)	.37	
	Right inferior temporal gyrus	0.72 (0.08)	0.68 (0.06)	.06	

^a^Analyses of covariance, with age as a covariate.

^b^ICV: intracranial volume.

### Correlation Between VR-Derived and MRI Biomarkers

**Figure 5:**
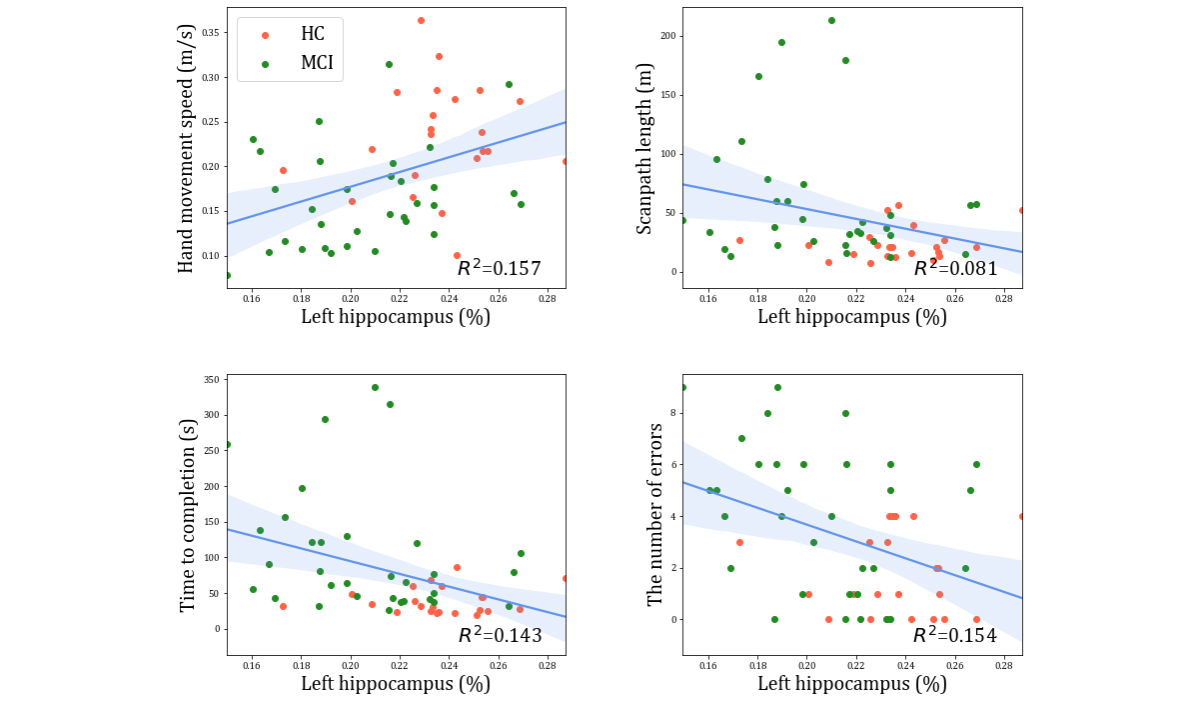
Significant correlations between 4 virtual reality–derived biomarkers (hand movement speed, scanpath length, time to completion, and the number of errors) and the magnetic resonance imaging biomarker, specifically, the left hippocampus. HC: healthy controls; MCI: mild cognitive impairment.

We conducted a Pearson correlation analysis to explore the relationships among statistically significant biomarkers, including VR-derived biomarkers (hand movement speed, scanpath length, time to completion, and the number of errors) and MRI biomarkers from the amygdala, hippocampus, and entorhinal cortex. The aim was to uncover any significant associations among these biomarkers and elucidate their potential implications within the context of this study. The analysis revealed several significant correlations. Hand movement speed correlated with the right amygdala (*r*=0.31; *P*=.02), left hippocampus (*r*=0.40; *P*=.003), right hippocampus (*r*=0.43; *P*=.002), left entorhinal cortex (*r*=0.32; *P*=.02), and right entorhinal cortex (*r*=0.35; *P*=.009). Scanpath length correlated significantly with the left hippocampus (*r*=−0.28; *P*=.04). Meanwhile, time to completion showed significant correlations with the left amygdala (*r*=−0.31; *P*=.02), left hippocampus (*r*=−0.38; *P*=.005), right hippocampus (*r*=−0.28; *P*=.04), left entorhinal cortex (*r*=−0.29; *P*=.04), and right entorhinal cortex (*r*=−0.29; *P*=.04). Furthermore, the number of errors demonstrated significant correlations with the left amygdala (*r*=−0.34; *P*=.01), right amygdala (*r*=−0.38; *P*=.005), left hippocampus (*r*=−0.39; *P*=.003), right hippocampus (*r*=−0.38; *P*=.005), left entorhinal cortex (*r*=−0.29; *P*=.03), and right entorhinal cortex (*r*=−0.34; *P*=.01). Notably, the left hippocampus exhibited significant correlations with all 4 VR-derived biomarkers (Figure 5).

### Multimodal Learning Performance Using Both VR-Derived and MRI Biomarkers

**Figure 6:**
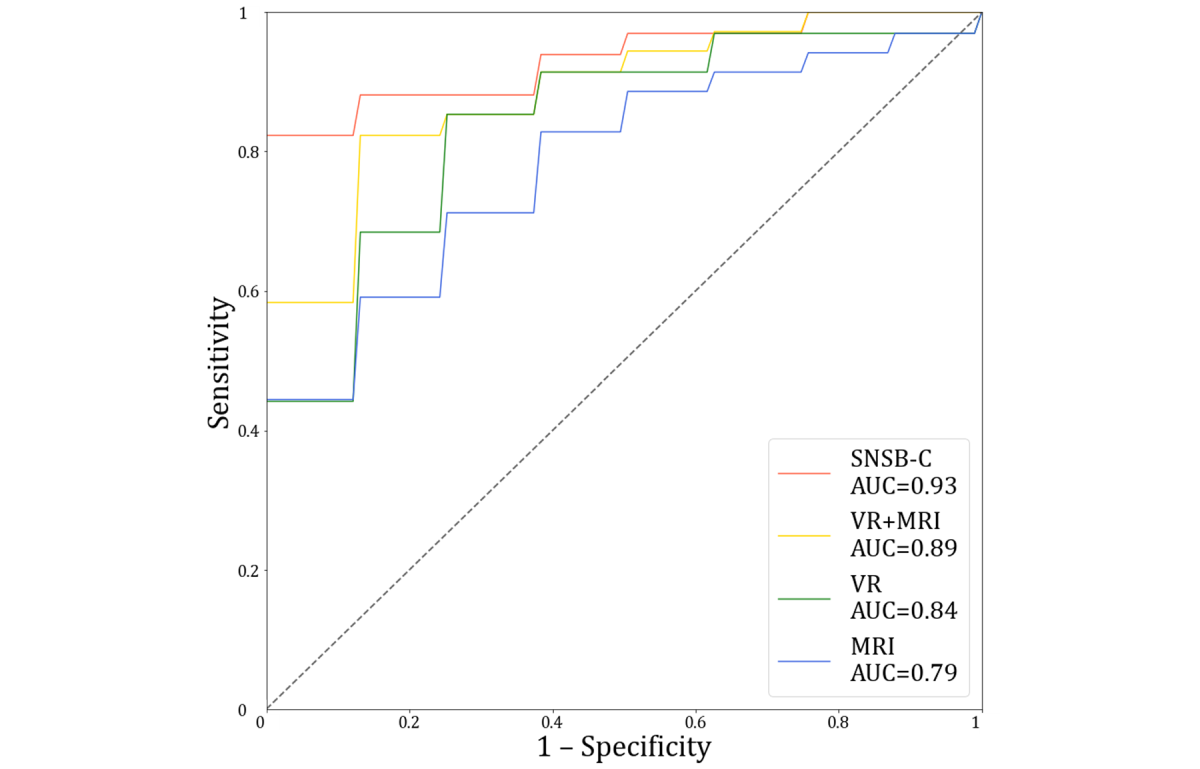
Comparison of receiver operating characteristic curves and the area under the receiver operating characteristic curve (AUC). The best classification performance was obtained when the support vector machine was trained using a combination of virtual reality (VR)–derived biomarkers (hand movement speed, scanpath length, and the number of errors) and magnetic resonance imaging (MRI) biomarkers (left entorhinal cortex and left hippocampus). The gold-standard Seoul Neuropsychological Screening Battery–Core (SNSB-C; Rey Complex Figure Test and Seoul Verbal Learning Test–Elderly’s Version–Delayed Recall) was omitted from this comparison.

The comparative performance of multimodal learning is presented in Table 5 and Figure 6. Initially, the SVM model trained with SNSB-C data (RCFT and SVLT-E–DR), considered as a gold standard for MCI classification, exhibited strong performance, achieving an accuracy of 94.4%, a sensitivity of 100%, a specificity of 85.7%, a precision of 91.7%, an *F*_1_-score of 95.7%, and an AUC of 0.93. The results shown in Table 5 indicate that combining SNSB-C results with either VR-derived or MRI biomarkers improves MCI classification performance compared to using VR or MRI alone. The best-performing SVM model using only VR-derived biomarkers, including hand movement speed, scanpath length, and the number of errors, achieved an accuracy of 88.9%, a sensitivity of 87.5%, a specificity of 90%, a precision of 87.5%, an *F*_1_-score of 87.5%, and an AUC of 0.84 (refer to Multimedia Appendix 2 for more details). In the unimodal SVM model relying solely on MRI biomarkers, the combination of the left hippocampus and left entorhinal cortex led to the best performance, with an accuracy of 83.3%, a sensitivity of 90.9%, a specificity of 71.4%, a precision of 83.3%, an *F*_1_-score of 87%, and an AUC of 0.79 (refer to Multimedia Appendix 3 for more details). Remarkably, the highest performance was achieved when both VR-derived and MRI biomarkers—specifically, hand movement speed, scanpath length, the number of errors, left entorhinal cortex, and left hippocampus—were integrated. This multimodal approach yielded an accuracy of 94.4%, a sensitivity of 100%, a specificity of 90.9%, a precision of 87.5%, an *F*_1_-score of 93.3%, and an AUC of 0.89, with the corresponding code accessible on GitHub [54]. These results suggest that a combined approach using both VR-derived and MRI biomarkers offers the most promising outcomes, closely resembling the gold standard represented by SNSB-C. In addition, the use of VR-derived biomarkers alone showed promising results, whereas the performance while using MRI biomarkers alone was relatively lower.

**Table 5:** Comparative performance of the multimodal learning approach using Seoul Neuropsychological Screening Battery–Core (SNSB-C), virtual reality (VR)–derived biomarkers, and magnetic resonance imaging (MRI) biomarkers used in the support vector machine model.

Biomarker data	Accuracy, %	Sensitivity, %	Specificity, %	Precision, %	*F*_1_-score, %
SNSB-C	94.4	100	85.7	91.7	95.7
SNSB-C+VR	94.4	92.3	100	100	96
SNSB-C+MRI	94.4	100	83.3	92.3	96
VR+MRI	94.4	100	90.9	87.5	93.3
VR	88.9	87.5	90	87.5	87.5
MRI	83.3	90.9	71.4	83.3	87

## Discussion

### Principal Findings

The primary objective of this study was to probe the effectiveness of a multimodal learning approach, integrating both VR-derived and MRI biomarkers, in augmenting early MCI detection. The obtained results showed significant differences in both VR-derived and MRI biomarkers when comparing patients with MCI and healthy controls. Specifically, patients with MCI displayed considerably slower hand movement speed, lengthier scanpath length, prolonged time to completion, and a greater number of errors in the virtual kiosk test compared to their healthy counterparts. The MRI biomarkers indicated noteworthy cerebral atrophy in the bilateral amygdala, hippocampus, and entorhinal cortex among patients with MCI. A remarkable observation was the superior performance of the multimodal learning approach, which incorporated both VR-derived and MRI biomarkers, in the prediction of MCI. This integrated approach achieved an accuracy of 94.4%, a sensitivity of 100%, a specificity of 90.9%, a precision of 87.5%, an *F*_1_-score of 93.3%, and an AUC of 0.89. It outperformed models based solely on either VR-derived or MRI biomarkers and showed performance comparable to that of SNSB-C, the gold standard assessment tool for MCI diagnosis in our study. Importantly, the combination of VR-derived and MRI biomarkers allowed for faster MCI detection, significantly reducing the time required compared to neuropsychological tests such as SNSB-C, which typically take approximately 2 hours [55]. These findings provide substantial evidence highlighting the potential advantages of using a combination of VR-derived and MRI biomarkers for enhancing the detection of MCI.

Our findings from the multimodal learning approach demonstrated the distinct advantages of VR-derived and MRI biomarkers in identifying patients with MCI. VR-derived biomarkers exhibited remarkable specificity of 90%, indicating their effectiveness in accurately classifying healthy controls. This finding aligns with previous studies demonstrating the high specificity of VR-derived biomarkers obtained through IADL tasks in VR, such as financial activities or public transportation tasks [16] and shopping in virtual supermarkets [56,57]. On the other hand, MRI biomarkers displayed superior sensitivity of 90.9%, showcasing their proficiency in correctly detecting patients with MCI. Interestingly, our results showed relatively higher sensitivity compared to recent MRI biomarker studies reviewed by Lombardi et al [58]. Recent MRI studies focused on optimizing a wide range of MRI features [59] and investigated subfields within brain regions, such as the hippocampal tail [60,61]. Meanwhile, our approach incorporated recent techniques for MCI screening. First, we separately measured the volumes of the left and right hemispheres to account for hemisphere asymmetry, as suggested by Mabrouk et al [62]. Subsequently, we used the hippocampus and entorhinal cortex as MRI biomarkers, which are well recognized for their sensitivity in MCI classification, based on the findings of Park et al [63]. Finally, to mitigate the potential influences of individual characteristics on brain volume, we applied the recently acclaimed ICV normalization technique [64,65]. We believe that these approaches may have contributed to the enhanced sensitivity in MCI detection compared to traditional methodologies. However, despite their strengths, each modality also has identifiable limitations. VR-derived biomarkers may sporadically misclassify patients with MCI as healthy controls, whereas MRI biomarkers might occasionally misidentify healthy controls as patients with MCI. By fusing the strengths of both VR-derived and MRI biomarkers, we managed to circumvent these restrictions, thereby achieving impressive rates of specificity (90.9%) and sensitivity (100%). These results underscore the potential benefits of multimodal learning, harnessing the complementarity of VR-derived and MRI biomarkers to significantly improve the early detection of MCI.

The distinctive advantages of VR-derived and MRI biomarkers suggest their potential use as sequential diagnostic tools in a 2-stage diagnostic process, as proposed by Galvin et al [66]: the “detection” and “assessment and differentiation” phases. In the detection phase, a swift screening tool can be used to identify the risk of MCI among a wider population of older adults. VR-derived biomarkers align well with the requirements for a rapid screening tool in this phase, offering a brief testing duration (<5 min) with high specificity. Thus, they are effective in distinguishing healthy controls and identifying potential patients with MCI who may require further evaluation. For MCI care, these VR-derived biomarkers can be implemented in local dementia care centers. When VR-derived biomarkers are used to identify the risk of MCI among residents in these centers, individuals can be referred to local hospitals for more specific dementia assessments. Subsequently, during the assessment and differentiation phase, individuals who are flagged as potential MCI cases in the detection phase can undergo a more thorough diagnostic process to confirm MCI. In this study, MRI biomarkers prove to be an apt choice, given their high sensitivity in diagnosing patients with MCI and their ability to offer clinical evidence regarding changes in brain structure. For MCI care, the results from MRI biomarkers can be used to accurately diagnose patients with MCI and tailor treatment plans. Furthermore, the integration of both VR-derived and MRI biomarkers demonstrated the highest MCI classification performance. Overall, integrating VR-derived biomarkers as a rapid screening tool into the detection phase and MRI biomarkers as a diagnostic tool into the assessment and differentiation phase can significantly enhance the early detection of and care process for MCI. This approach not only reduces time and cost burdens on individuals but also provides invaluable support to clinicians in making accurate MCI diagnoses for the local older adult population.

**Figure 7:**
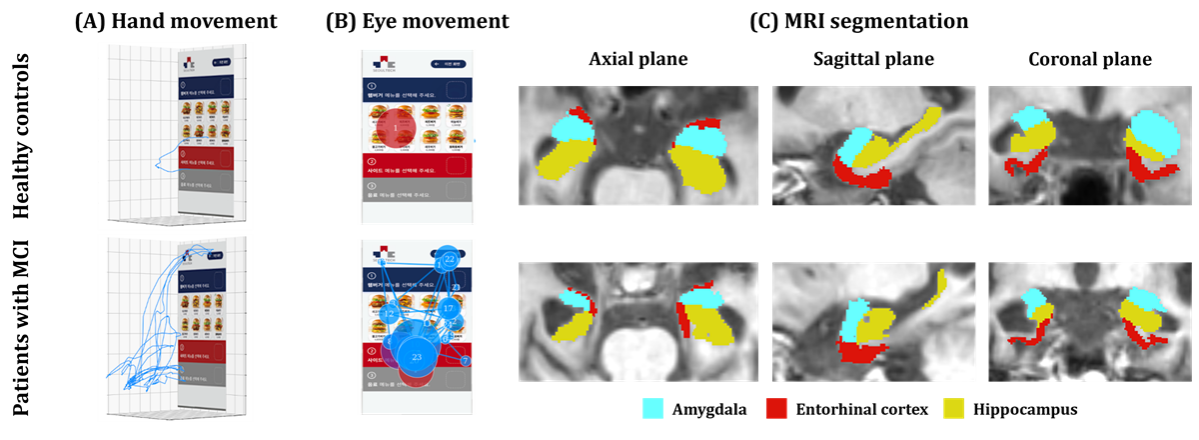
Comparison of hand movements, eye movements, and T1-weighted magnetic resonance imaging (MRI) between healthy controls and patients with mild cognitive impairment (MCI). (A) 3D coordinates of hand movements (depicted in blue). (B) Participant focus points indicated by dots, with red, blue, and purple representing the start, middle, and end of gaze, respectively—dot size corresponds to fixation duration. (C) Patients with MCI exhibiting statistically significant atrophy in the amygdala, hippocampus, and entorhinal cortex compared to healthy controls.

Our study’s findings highlight a notable correlation between behavioral characteristics derived from VR-derived biomarkers and observed alterations or damage in the brain, as identified through MRI biomarkers. The correlation analysis unveiled a positive relationship between VR-derived biomarkers and the left hippocampus. This implies that participants with a larger left hippocampus tended to exhibit faster hand movement speed, shorter scanpath length, reduced time to completion, and fewer errors during the virtual kiosk test. Considering the pivotal role of the hippocampus in memory formation and learning, this observation is congruent with previous studies [67,68] suggesting that hippocampal damage may lead to cognitive deficits and impact behaviors, including hand and eye movements during everyday activities. Consequently, individuals with hippocampal damage may display complex hand and eye movements when performing tasks such as those presented in the virtual kiosk test (as illustrated in Figure 7). An intriguing finding is that eye movement features exhibited significant correlations with MRI biomarkers (ie, the left hippocampus) and SNSB-C measures (ie, S-K–BNT, RCFT, and DSC), which aligns with recent studies emphasizing the potential importance of eye movements in identifying MCI during complex daily tasks [69,70]. Simultaneously, negative correlation was observed between the number of errors and the right amygdala, implying that participants with a smaller right amygdala tended to make more errors during the virtual kiosk test. The amygdala also plays a crucial role in memory, consistent with the findings of earlier studies [71,72] linking volume reduction to cognitive impairment. Consequently, individuals with amygdala damage may be more prone to errors during task performance. In this context, the use of VR-derived biomarkers obtained from augmented reality or VR tests involving daily activities represents a pioneering approach for the early detection of MCI [73,74], bridging the gap between the identification of behavioral abnormalities and the underlying structural changes in the brain [75,76].

This study had certain limitations that should be acknowledged. First, the MCI classification model in this study did not encompass a diverse range of individuals with varying racial characteristics [77] or include those with neurodegenerative diseases such as Parkinson disease [78] and Lewy body diseases [79], which could potentially impact cognitive impairment in older adults. Future studies investigating these aspects may aid in the development of screening tools for neurodegenerative diseases among older adults, expanding the potential user pool to include different racial groups. Second, while this study used handcrafted features extracted from VR-derived biomarkers, training deep learning models directly on the input data could allow for a more comprehensive analysis of patterns and the exploration of novel features. Finally, although SVM was used as the multimodal learning approach based on previous studies [4,48-50], alternative multimodal learning approaches should be explored and compared to potentially improve classification performance. For instance, Contrastive Language-Image Pretraining [80] is one of the multimodal integration models using text and images. Similarly, future multimodal research with VR-derived and MRI biomarkers is necessary to delve deeper into their relationship.

Despite the limitations acknowledged, our study makes a noteworthy contribution to the field compared to previous studies [21,23]. This is achieved by attaining superior MCI detection performance through the integration of VR-derived and MRI biomarkers via multimodal learning, surpassing the performance using VR-derived or MRI biomarkers individually. This consolidated approach garnered remarkable results, such as an accuracy of 94.4%, a sensitivity of 100%, a specificity of 90.9%, a precision of 87.5%, an *F*_1_-score of 93.3%, and an AUC of 0.89. These findings underscore the synergistic benefits of integrating VR-derived and MRI biomarkers. Moreover, our correlation analysis between VR-derived and MRI biomarkers illustrated how structural brain changes can translate into behavioral modifications in daily life. Finally, we proposed an innovative clinical application strategy, wherein VR-derived biomarkers are used in the preliminary detection stage, followed by MRI biomarkers in the assessment and differentiation stage. With a high specificity (90%) being ideal for initial screening, VR-derived biomarkers are perfect for the first stage, whereas MRI biomarkers, with their high sensitivity (90.9%), are optimal for the subsequent stage. This sequential approach could potentially reduce the time and cost burden for individuals and provide clinicians valuable assistance in making accurate diagnoses. To summarize, our study demonstrated the advantages of VR-derived and MRI biomarkers’ integration using multimodal learning to enhance early MCI detection.

### Conclusions

This study determined the unique characteristics of VR-derived and MRI biomarkers while highlighting the significance of integrating both biomarkers for early detection of MCI. The results imply that selecting the appropriate biomarker at different stages is beneficial. Specifically, VR-derived biomarkers with high specificity can be used as an early screening tool for MCI, whereas MRI biomarkers with high sensitivity are more suitable for confirming MCI. However, the most valid approach is integrating both VR-derived and MRI biomarkers, as the SVM trained using both biomarkers outperformed other models that used a single biomarker. These compelling results provide strong evidence for the potential of multimodal learning to enhance overall diagnostic performance in the early detection of MCI. Furthermore, our study contributes to understanding how structural brain changes can manifest as behavior changes by showing the relationship between VR-derived and MRI biomarkers. Our study’s multimodal learning approach offers valuable insights into enhancing early MCI detection by integrating diverse biomarkers.

## Appendix

Multimedia Appendix 1 Comparison of raw volumes of magnetic resonance imaging (MRI) biomarkers between healthy controls and patients with mild cognitive impairment (MCI).

Multimedia Appendix 2 Comparative performance of different virtual reality (VR)–derived feature combinations utilized in the Support Vector Machine (SVM) model.

Multimedia Appendix 3 Comparative performance of different magnetic resonance imaging feature combinations used in the support vector machine model.

## Abbreviations

AD: Alzheimer disease
ANCOVA: analysis of covariance
AUC: area under the receiver operating characteristic curve
IADL: instrumental activities of daily living
ICV: intracranial volume
MCI: mild cognitive impairment
MRI: magnetic resonance imaging
RCFT: Rey Complex Figure Test
SNSB-C: Seoul Neuropsychological Screening Battery–Core
SVM: support vector machine
VR: virtual reality

## Data Availability

The data sets produced and examined in this study are not publicly accessible due to concerns related to the privacy and consent of research participants. All data and materials related to the study are securely stored at the Department of Applied Artificial Intelligence, Seoul National University of Science and Technology, and can be accessed upon request.
