# Supplementary material for: Integrating Biomarkers From Virtual Reality and Magnetic Resonance Imaging for the Early Detection of Mild Cognitive Impairment Using a Multimodal Learning Approach: Validation Study
Source: J Med Internet Res. 2024 Apr 17;26:e54538. doi: 10.2196/54538 (PMC11063880; doi:10.2196/54538)
Supplement: Multimedia Appendix 1 [file jmir_v26i1e54538_app1.docx]

Table S1. Comparison of raw volumes of magnetic resonance imaging (MRI) biomarkers between healthy controls and patients with mild cognitive impairment (MCI).

| MRI biomarkers | | Group, mean (SD) | | *P* value^a^ |
| --- | --- | --- | --- | --- |
|  | | Healthy controls (n=22) | Patients with MCI (n=32) |  |
|  | | | | |
| **Raw volume (cc)** | | | | |
|  | ICV^b^ | 1490.96 (127.40) | 1511.49 (128.89) | .56 |
|  | Left cerebral white matter | 214.66 (19.94) | 212.75 (24.62) | .74 |
|  | Right cerebral white matter | 216.21 (20.12) | 213.70 (24.72) | .82 |
|  | Left cerebral gray matter | 230.68 (15.22) | 226.33 (23.65) | .76 |
|  | Right cerebral gray matter | 229.80 (16.88) | 223.26 (24.17) | .49 |
|  | Left ventricles | 19.76 (13.04) | 24.01 (13.90) | .59 |
|  | Right ventricles | 18.05 (14.93) | 21.60 (12.65) | .65 |
|  | Left amygdala | 1.68 (0.15) | 1.57 (0.22) | .05 |
|  | Right amygdala | 1.82 (0.14) | 1.67 (0.26) | .01 |
|  | Left hippocampus | 3.50 (0.30) | 3.11 (0.52) | .008 |
|  | Right hippocampus | 3.58 (0.29) | 3.29 (0.52) | .037 |
|  | Left entorhinal cortex | 2.25 (0.26) | 2.02 (0.34) | .024 |
|  | Right entorhinal cortex | 1.87 (0.18) | 1.71 (0.29) | .031 |
|  | Left parahippocampal gyrus | 1.96 (0.26) | 1.85 (0.25) | .28 |
|  | Right parahippocampal gyrus | 1.82 (0.22) | 1.76 (0.20) | .41 |
|  | Left fusiform gyrus | 8.98 (1.14) | 8.81 (1.05) | .76 |
|  | Right fusiform gyrus | 8.48 (1.40) | 8.57 (1.21) | .64 |
|  | Left superior temporal gyrus | 10.67 (1.02) | 10.38 (1.37) | .69 |
|  | Right superior temporal gyrus | 10.22 (0.69) | 10.14 (1.21) | .77 |
|  | Left middle temporal gyrus | 10.48 (1.13) | 9.98 (1.48) | .34 |
|  | Right middle temporal gyrus | 10.22 (1.05) | 9.81 (1.52) | .32 |
|  | Left inferior temporal gyrus | 11.23 (1.01) | 11.16 (1.33) | .92 |
|  | Right inferior temporal gyrus | 10.72 (1.22) | 10.26 (1.28) | .30 |

^a^Analyses of covariance, with age as a covariate.

^b^ICV: intracranial volume.
