# Supplementary material for: Integrating Biomarkers From Virtual Reality and Magnetic Resonance Imaging for the Early Detection of Mild Cognitive Impairment Using a Multimodal Learning Approach: Validation Study
Source: J Med Internet Res. 2024 Apr 17;26:e54538. doi: 10.2196/54538 (PMC11063880; doi:10.2196/54538)
Supplement: Multimedia Appendix 2 [file jmir_v26i1e54538_app2.docx]

Table S1. Comparative performance of different virtual reality (VR)–derived feature combinations utilized in the Support Vector Machine (SVM) model.

| Combinations of features | Accuracy, % | Sensitivity, % | Specificity, % | Precision, % | F1 score, % |
| --- | --- | --- | --- | --- | --- |
| Hand movement speed + Scanpath length + The number of errors | 88.9 | 87.5 | 90.0 | 87.5 | 87.5 |
| Scanpath length + Time to completion + The number of errors | 83.3 | 83.3 | 83.3 | 90.9 | 87.0 |
| Hand movement speed + The number of errors | 83.3 | 81.8 | 85.7 | 90.0 | 85.7 |
| Hand movement speed + Scanpath length | 77.8 | 77.8 | 77.8 | 77.8 | 77.8 |
| Scanpath length + The number of errors | 77.8 | 72.7 | 85.7 | 88.9 | 80.0 |
