# Supplementary material for: Integrating Biomarkers From Virtual Reality and Magnetic Resonance Imaging for the Early Detection of Mild Cognitive Impairment Using a Multimodal Learning Approach: Validation Study
Source: J Med Internet Res. 2024 Apr 17;26:e54538. doi: 10.2196/54538 (PMC11063880; doi:10.2196/54538)
Supplement: Multimedia Appendix 3 [file jmir_v26i1e54538_app3.docx]

Table S1. Comparative performance of different magnetic resonance imaging (MRI) feature combinations utilized in the Support Vector Machine (SVM) model.

| Combinations of features | Accuracy, % | Sensitivity, % | Specificity, % | Precision, % | F1 score, % |
| --- | --- | --- | --- | --- | --- |
| Left hippocampus + Left entorhinal cortex | 83.3 | 90.9 | 71.4 | 83.3 | 87.0 |
| Left hippocampus + Right hippocampus | 88.3 | 88.9 | 77.8 | 80.0 | 84.2 |
| Left entorhinal cortex + Right amygdala | 77.8 | 88.9 | 66.7 | 72.7 | 80.0 |
| Left entorhinal cortex + Right hippocampus | 77.8 | 81.8 | 71.4 | 81.8 | 81.8 |
| Right entorhinal cortex + Right amygdala | 72.7 | 81.8 | 57.1 | 75 | 78.3 |
